# The tip of the iceberg: Rethinking burnout through social and organisational lenses

**DOI:** 10.1111/medu.70034

**Published:** 2025-09-10

**Authors:** Megan E. Kruskie, Jessica N. Byram, Kyle A. Robertson

**Affiliations:** ^1^ Department of Biochemistry and Molecular & Cellular Biology Georgetown University School of Medicine Washington DC USA; ^2^ Department of Anatomy, Cell Biology, & Physiology Indiana University School of Medicine Indianapolis Indiana USA; ^3^ Department of Anatomy, Cell Biology, & Physiology Indiana University School of Medicine West Lafayette Indiana USA

## Abstract

Kruskie et al. offer argument contending that framing burnout in the contexts of organizational and socialization theories can help us better understand the phenomenon.

In this issue of *Medical Education*, Prentice and colleagues present the results of a systematic review and meta‐analysis of burnout in postgraduate medical trainees before and after the COVID‐19 pandemic while also considering specialty, gender, training level and regional differences. They primarily included studies based on the Maslach Burnout Inventory (MBI), the most frequently utilised instrument to ‘measure’ burnout.^1^ In this commentary, we seek to challenge conventional thinking about burnout, its conceptualisation and measurement. We argue that framing burnout in the contexts of organisational and socialisation theories may help us better understand the phenomenon.

Framing burnout in the contexts of organisational and socialisation theories may help us better understand the phenomenon.

Although this is a commentary, we feel it is important that readers understand the philosophical foundations from which we approach this work. There is a running controversy in the field about whether burnout is a state, a condition exacerbated by organisational demands and lack of resources, or a trait, a stable characteristic of a person that influences their behaviour across time. We contend that burnout is a state but also that individuals are active players who can impact their work environment through job crafting.

We contend that burnout is a state but also that individuals are active players who can impact their work environment through job crafting.

A chief concern when conducting meta‐analyses of burnout is the heterogeneity of definitions. For example, burnout is defined by the World Health Organization as a syndrome resulting from chronic workplace stress that has not been successfully addressed. This definition has three characteristic dimensions: ‘feelings of energy depletion or exhaustion; increased mental distance from one's job, or feelings of negativism or cynicism related to one's job; and reduced professional efficacy’.^2^ Conversely, the 11th revision of the International Classification of Diseases (ICD‐11) defines burnout as an occupational phenomenon, not as a syndrome or medical condition. Considering the vast heterogeneity of experiences and the array of non‐specific symptoms that are claimed as characteristic, standardising diagnostic criteria for burnout as a ‘syndrome’ is difficult, if not impossible.^3^

A brief timeline of the evolution of burnout starts with Freudenberger who coined the term in the 1970s. He suggested that burnout is a highly individual phenomenon that ‘reflects the experience of symptoms of any kind, which are experienced as a result of overload’.^4,p8^ Despite its early description, the current study^1^ approaches burnout from an epidemiological perspective that favours its syndromic interpretation. We would argue that this approach, which is consistent with a biomedical model of symptoms and disease, has the effect of pathologising the individual and their experiences as a problem to be fixed. By contrast, a phenomenological perspective, which we favour, focuses on the lived experience and meaning created by the individual and, in this way, is less likely to lead to stigmatisation and the need for treatment.

A recent review by Canu and colleagues^5^ used semantic analysis and a Delphi method to attempt to harmonise the no fewer than 13 extant iterations and conceptualisations of burnout, ranging from Freudenberger (1974) to Schaufeli and colleagues' (2019) work on a Burnout Assessment Tool. In doing so, they provide the following definition that supports burnout as a state (phenomenon) rather than a trait (syndrome):

In a worker, occupational burnout or occupational physical AND emotional exhaustion state is an exhaustion due to prolonged exposure to work‐related problems.^5,p104^

The fluid definition and methodological heterogeneity employed in studying burnout make it challenging to meaningfully interpret its prevalence. Some argue this may be due, in part, to how the MBI itself conceptualises burnout.^3^, ^6^ Importantly, by some estimates, the MBI has been used in some 90% of burnout studies. At the same time, there have been critiques of how the MBI treats burnout as one concept but measures it in three dimensions, bringing into question the relationship between the concept and its measurement.^6^ We are left asking, why is there such controversy surrounding the conceptualisation and definition of burnout? Is it rooted in the complexity of defining what counts as burnout and differing philosophical frameworks? Or is it such a deeply rooted part of the human experience that attempts to measure burnout lose its meaning for the individual?

We are left asking, why is there such controversy surrounding the conceptualisation and definition of burnout?

Informing and closely related to burnout is research on stress. To date, research in this domain has been dominated by a cognitive psychological conceptualisation focusing on how individuals respond to their environment with minimal consideration of the contextual and environmental influences that shape and affect behaviour. An alternative viewpoint embeds the individual in the culture or context, in and through which the repertoire of responses to the environment is learned from joint interactions with others.^7^

Inherent in this individualistic paradigm is to measure stress, and consequently burnout, using self‐reported data that is more in line with ‘appraisals of individuals, not individual appraisal’.^7,p32^ Hobfoll uses the apt iceberg analogy where the self‐report portion is the iceberg above the water line, but below the water line influencing these data are the individuals and their biases, based on interpreted familial and cultural norms (see fig 2.1 in Hobfoll^7,p33^). Coming back to burnout research, we must use this evolution of the stress literature to consider if we are only viewing the tip of the iceberg by using self‐reported questionnaire data, missing the underlying influences.

Are only viewing the tip of the iceberg by using self‐reported questionnaire data, missing the underlying influences?

A proposed approach is to ground burnout research in a contextual manner by exploring it through an integrated theoretical framework utilising job demands‐resources (JD‐R) theory, conservation of resources (COR) theory, belonginess and self‐determination theory (SDT).^7^, ^8^, ^9^, ^10^ To do so may facilitate a deeper and more nuanced understanding of the phenomenon. Combining these into a unified or integrated interpretative framework would be to appreciate social context, not merely as background noise, but as actively shaping resource availability, identity and motivation. In this framing, reductions in stress and improvements in well‐being would be optimised when individuals have sufficient resources, manageable demands and a secure, valued identity.

Reductions in stress and improvements in well‐being would be optimised when individuals have sufficient resources, manageable demands and a secure, valued identity.

Our further suggestion, based on the current state of research on burnout, is to shift the paradigm from an individualistic perspective to an integrated ‘individual‐nested‐in family‐nested‐in social organization’ framework in the hopes of appreciating a more holistic and nuanced understanding.^7,p55^ We look forward to helping and observing the field continue to move in such directions.

## AUTHOR CONTRIBUTIONS

**Megan E. Kruskie:** Conceptualization; writing—original draft; writing—review and editing. **Jessica N. Byram:** Conceptualization; writing—original draft; writing—review and editing. **Kyle A. Robertson:** Conceptualization; writing—original draft; writing—review and editing.
